# The complete chloroplast genome of *Osmanthus serrulatus* rehd., a natural sweet olive endemic to China

**DOI:** 10.1080/23802359.2019.1674733

**Published:** 2019-10-07

**Authors:** Lin Chen, Longna Li, Tingting Pan, Xiangui Yi, Xianrong Wang, Mingzhi Li

**Affiliations:** a Co-Innovation Center for Sustainable Forestry in Southern China, Nanjing Forestry University, Nanjing, China;; b International Cultivar Registration Center for Osmanthus, College of Biology and the Environment, Nanjing Forestry University, Nanjing, China;; c College of Life Sciences, Laboratory Center of Life Sciences, Nanjing Agricultural University, Nanjing, China;; d Biodata Biotechnologies Co. Ltd, Hefei, China

**Keywords:** *Osmanthus serrulatus*, chloroplast genome, phylogenetic analysis, Oleaceae

## Abstract

*Osmanthus serrulatus* is an endemic tree in southwest China. Here, the complete chloroplast genome of this species was assembled and characterised from high-throughput data. The entire circular genome of *O. serrulatus* was 155,465 bp in size, which composed of large single-copy (LSC) and small single-copy (SSC) regions of 86,545 bp and 17,494 bp, respectively, and separated by a pair of inverted repeat (IR) regions of 25,713 bp each. The genome contained 133 genes, including 88 protein-coding genes, 37 tRNA genes, and eight rRNA genes. The overall GC content of the genome is 37.8%. A phylogenetic tree reconstructed by 35 chloroplast genomes reveals that *O. serrulatus* is most related with *O. yunnanensis*.

*Osmanthus serrulatus* Rehd. (Oleaceae), usually known as Baoxing osmanthus, is one of the rare spring flowering endemic *Osmanthus* species of which narrowly and sporadically distributed under the evergreen broad-leaved forests at elevations of 1800–2400 m in west Sichuan Province, China (Xiang and Liu 2008). Although it occupies an important phylogenetic node in genus *Osmanthus*, and plays a crucial germplasm resource for genetic breeding of sweet osmanthus, the genetic information of *O. serrulatus* is still limited so far (Chen et al. 2015), while most study always focus on *O. fragrans* (Mu et al. 2014; Yang et al. 2018; Duan et al. 2019). So, it is necessary to develop genetic resources of *O. serrulatus* to provide basic intragenic information for the further study on phylogeny and biogeography in genus *Osmanthus*.

The fresh leaves of *O. serrulatus* were sampled from Baoxing, Sichuan, China (102°40′41″E, 30°24′18″N). Specimens were stored in the Herbarium of Nanjing Forestry University with accession number of NF20190175. Total genomic DNA was extracted with a modified CTAB protocol according to Doyle and Doyle (1987). The whole genome sequencing was conducted by Hefei Biodata Biotechnologies Inc. (Hefei, China) on the Illumina Hiseq platform. The filtered sequences were assembled using the programme SPAdes assembler 3.10.0 (Anton et al. 2012). Annotation was performed using the DOGMA (Wyman et al. 2004) and BLAST searches.

The cp genome of *O. serrulatus* was determined to comprise a 155,465 bp double stranded, circular DNA (GenBank accession no. MN480301), which containing two inverted repeat (IR) regions of 25,713 bp, separated by large single-copy (LSC) and small single-copy (SSC) regions of 86,545 bp and 17,494 bp, respectively. The overall GC content of *O. serrulatus* cp genome is 37.8% and the corresponding values in LSC, SSC and IR regions are 35.8%, 32.0% and 43.2%, respectively. The cp genome was predicted to contain 133 genes, including 88 protein-coding genes, 37 tRNA genes, and 8 rRNA genes. Seven protein-coding genes, seven tRNA genes and four rRNA genes were duplicated in IR regions. Nineteen genes contained two exons and four genes (*clpP*, *ycf3* and two *rps12*) contained thee exons.

To investigate its taxonomic status and phylogenetic position, alignment was performed on the 35 chloroplast genome sequences using MAFFT v7.307 (Kazutaka and Standley 2013), and a maximum likelihood (ML) tree was constructed by FastTree version 2.1.10 (Price et al. 2010). As expected, *O. serrulatus* is mostly related to *O. yunnanensis*, with bootstrap support values of 99% (Figure 1). The complete cp genome sequence of *O. serrulatus* will provide a useful resource for the conservation genetics of this species as well as for the phylogenetic studies of Oleaceae.

**Figure 1.**
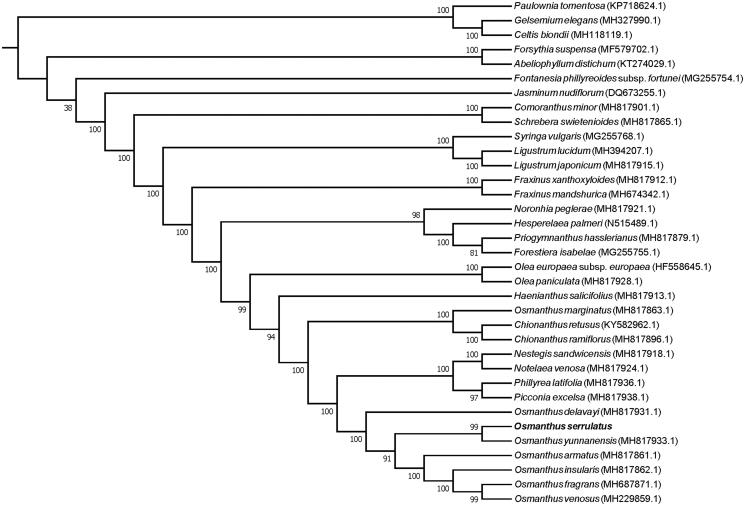
Phylogenetic tree inferred by maximum-likelihood (ML) method based on the complete chloroplast genome of 32 representative species of Oleaceae, with 3 species from Lamiales as outgroup.
